# Prognostic value of AKT isoforms in non-small cell lung adenocarcinoma

**DOI:** 10.7555/JBR.36.20220138

**Published:** 2022-11-10

**Authors:** Sahil Khurana, Ajay Pal Singh, Ashok Kumar, Rajeev Nema

**Affiliations:** 1 All India Institute of Medical Sciences Bhopal, India 462021; 2 Department of Medicine, All India Institute of Medical Sciences Virbhadra Road, Rishikesh Uttarakhand, India 249201; 3 Department of Biochemistry, All India Institute of Medical Sciences AIIMS Bhopal, 462021; 4 Department of Oncology, 3B Black Bio Biotech India Ltd. 7-C, Industrial Area, Govindpura, Bhopal, India 462023

Dear Editor,

Lung cancer is one of the most prevalent cancers in the world and has a high mortality rate. Lung cancer patients often have a poor prognosis, with a five-year survival rate of only about 16%. The International Agency for Research on Cancer reports that lung cancer was the main cause of cancer deaths in 2020, accounting for 1.80 million deaths. Due to the dismal overall prognosis of lung cancer, there is an urgent need to develop accurate and effective diagnostic tests that target specifically early oncogenic pathways in lung cancer patients to improve their prognosis.

The two principal types of lung cancer are small cell lung carcinoma (SCLC) and non-small cell lung carcinoma (NSCLC), with NSCLC accounting for around 85% of all lung malignancies^[1]^. The region frequency and prevalence of the lung disease are controlled by both genotypic and phenotypic exposures. An accurate lung cancer diagnosis is essential for the patient's better survival for two main reasons: appropriate drug selection and effective treatment prediction. Histopathological diagnosis depends on cell shape and the nucleus-to-cytoplasm size ratio to distinguish SCLC from NSCLC. Surgical resection, aggressive or palliative radiation, and neoadjuvant chemotherapy are frequently used to treat lung cancer. In the modern era, gene-targeted treatments against tyrosine kinase inhibitors and antibodies against mutations in driver genes for lung cancer are being developed. Several mutations have been shown to be the most common in lung cancer. For example, mutations in the K-ras proto-oncogene cause 10% to 30% of lung adenocarcinoma (LUAD), while epidermal growth factor receptor mutations are more frequent in squamous cell lung cancer (SqCLC)^[2]^.

Kaplan-Meier plotter (KM plotter) database (http://kmplot.com/analysis/) is a commonly used database for the real-time meta-analysis of published lung cancer microarray datasets to find survival biomarkers^[3]^. In a number of malignancies, the KM plotter has also been used to discover genes that may serve as possible prognostic indicators for post-progression survival (PPS), progression-free survival (PFS), and overall survival (OS)^[3]^. Many human malignancies, including lung cancer, have activated and overexpressed AKT isoforms^[4]^. AKT2 inhibition aids in the suppression of LUAD cell proliferation and colony expansion. Therefore, the significance of AKT isoforms in the diagnosis and prognosis of lung cancer was investigated in the current study. The association between gene specific mRNA expression and OS was analyzed by the KM plotter. Currently, gene expression and survival data from 1927 patients with a follow-up period of 20 years are available. Gene names of *AKT* isoforms (*i.e.*, *AKT1*, *AKT2*, and *AKT3*) were entered into the KM plotter database to obtain survival plots. The association between mRNA expression levels of different *AKT* isoforms and the established clinicopathological features was studied. The patient data were linked to OS as well as the sex of the patients.

We found that high *AKT1* mRNA expression was not significantly associated with OS in patients with lung cancer (hazard ratio [HR], 1.12; 95% confidence interval [CI], 0.99–1.27, *P*=0.071) or patients with SqCLC (HR, 0.84; 95% CI, 0.67–1.07; *P*=0.16), but was significantly associated with a poor OS in patients with LUAD (HR, 1.67; 95% CI, 1.31–2.11; *P*=2.1e−05 (***Fig. 1A***–***C***). High *AKT2* mRNA expression was also substantially associated with a poor OS in patients with lung cancer (HR, 1.67; 95% CI, 1.41–1.97; *P*=1.2e−09) and patients with LUAD (HR, 2.35; 95% CI, 1.82–3.02; *P*=9.7e−12), but not in patients with SqCLC (HR, 1.35; 95% CI, 0.98–1.86; *P*=0.062) (***Fig. 1D***–***F***). Furthermore, high *AKT3* mRNA expression was associated with a poor OS in patients with lung cancer (HR, 1.31; 95% CI, 1.16–1.49; *P*=2.2e−05) and patients with LUAD (HR, 2.08; 95% CI, 1.64–2.64; *P*=7.9e−10), but not in patients with SqCLC (HR, 0.97; 95% CI, 0.77–1.23; *P*=0.82) (***Fig. 1G***–***I***).

**Figure 1:**
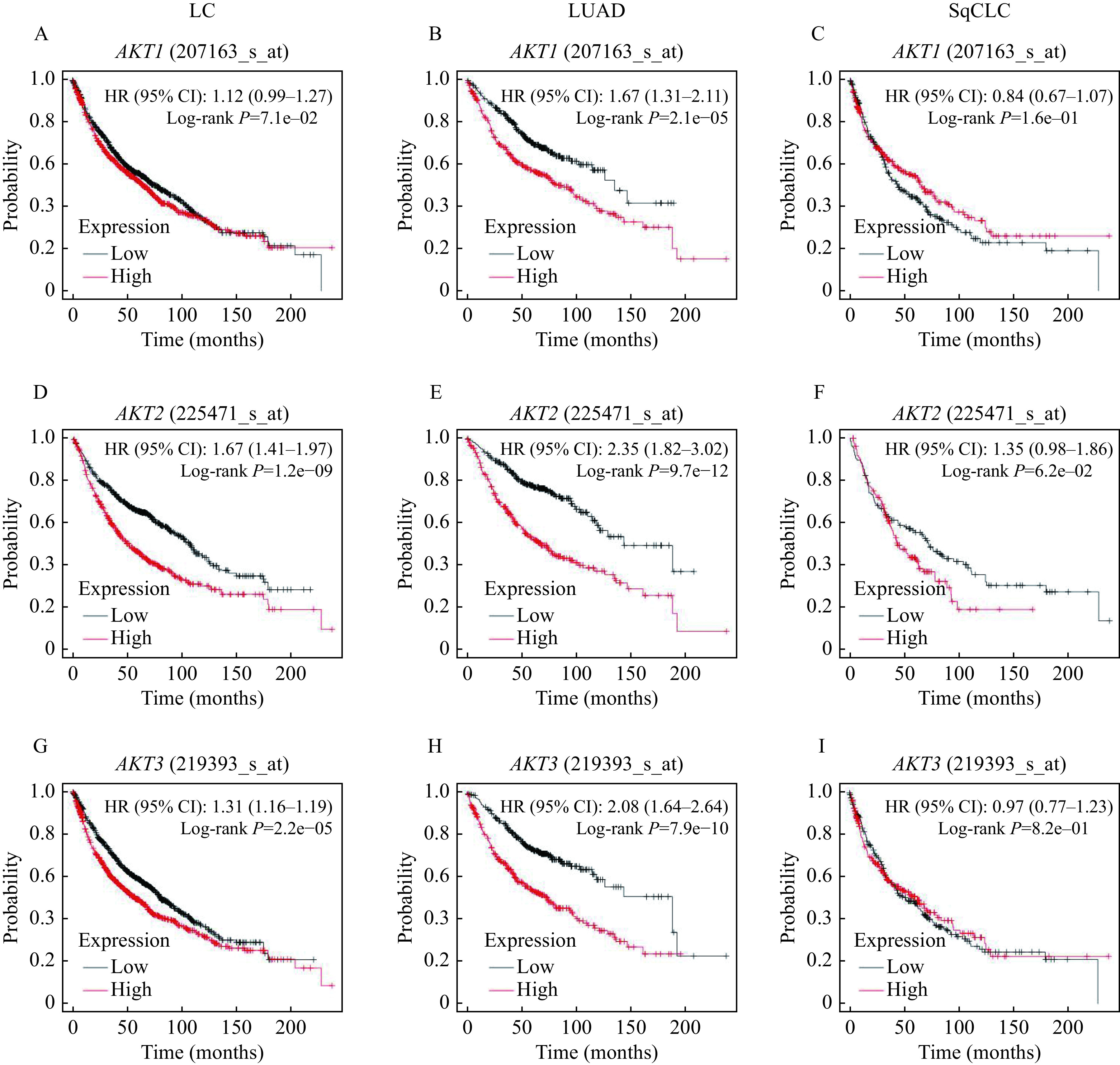
Prognostic value of AKT isoforms mRNA expression in non-small cell lung adenocarcinoma.

***Table 1*** shows that high expression of all AKT isoforms was significantly associated with a reduced PFS, that high expression of *AKT2* and *AKT3* was significantly associated with a reduced OS, and that high expression of *AKT2* was significantly associated with a reduced FPS. While analyzing the smoking history of the patients, high expression of *AKT3* was associated with a poor prognosis and a reduced OS regardless of smoking history (***Table 2***). ***Table 3*** shows the relevance of these genes with OS in terms of staging. High expression of *AKT1*, *AKT2* and *AKT3* was significantly associated with a OS in patients with clinical stage Ⅰ lung cancer, and high expression of *AKT3* was also found to be associated with a reduced OS in patients with clinical stage Ⅱ lung cancer. ***Table 4*** shows that high expression of *AKT2* and *AKT3* were associated with a reduced OS in patients regardless of sex; however, high expression of *AKT1* mRNA was not significantly associated with OS in either male or female lung cancer patients.

**Table 1:** Correlation between mRNA expression levels of *AKT* isoforms and survival outcomes in lung caner patients

Isoforms	Progression-free survival					Overall survival					Post-progression survival			
	Low (*N*)	High (*N*)	HR (95% CI)	*P*-value		Low (*N*)	High (*N*)	HR (95% CI)	*P*-value		Low (*N*)	High (*N*)	HR (95% CI)	*P*-value
*AKT1*	491	491	1.56 (1.29–1.9)	5.4e−06		964	961	1.12 (0.99–1.27)	7.1e−02		172	172	1.22 (0.95–1.57)	1.3e−01
*AKT2*	298	298	1.38 (1.05–1.81)	2.0e−02		572	572	1.67 (1.41–1.97)	1.2e−9		69	69	2.10 (1.36–3.24)	6.1e−04
*AKT3*	491	491	1.63 (1.34–1.98)	6.0e−07		966	959	1.31 (1.16–1.49)	2.2e−5		966	959	1.11 (0.87–1.44)	4.0e−01
The mRNA levels of *AKT* isoforms were classified into low and high expression groups according to the median value. Hazard ratio (HR) indicates the measure of the magnitude of the difference between the two curves from Kaplan-Meier plotter. CI: confidence interval.														

**Table 2:** Correlation between mRNA expression levels of *AKT* isoforms and overall survival of lung cancer patients with or without smoking history

Isoforms	Smoking					Non-smoking			
	Low (*N*)	High (*N*)	HR (95% CI)	*P*-value		Low (*N*)	High (*N*)	HR (95% CI)	*P*-value
*AKT1*	102	103	1.15 (0.94–1.42)	1.8e−01		411	409	2.48 (1.37–4.5)	2.0e−03
*AKT2*	70	71	1.89 (1.24–2.88)	2.7e−03		150	150	1.21 (0.54–2.71)	6.4e−01
*AKT3*	102	103	1.32 (1.07–1.62)	8.8e−03		411	409	2.28 (1.26–4.13)	5.3e−03
The mRNA levels of *AKT* isoforms were classified into low and high expression groups according to the median value. Hazard ratio (HR) indicates the measure of the magnitude of the difference between the two curves from Kaplan-Meier plotter. CI: confidence interval.									

**Table 3:** Correlation between mRNA expression levels of *AKT* isoforms and overall survival of stage **Ⅰ** and **stage Ⅱ** lung cancer patients

Isoforms	Stage Ⅰ					Stage Ⅱ			
	Low (*N*)	High (*N*)	HR (95% CI)	*P*-value		Low (*N*)	High (*N*)	HR (95% CI)	*P*-value
*AKT1*	288	289	1.76 (1.34–2.32)	4.5e−05		122	122	1.36 (0.94–1.96)	1.0e−01
*AKT2*	225	224	2.53 (1.81–3.52)	1.5e−08		82	79	1.5 (0.95–2.37)	7.7e−02
*AKT3*	290	287	2.2 (1.67–2.91)	1.2e−08		125	119	1.69 (1.17–2.44)	4.4e−04
The mRNA levels of *AKT* isoforms were classified into low and high expression groups according to the median value. Hazard ratio (HR) indicates the measure of the magnitude of the difference between the two curves from Kaplan-Meier plotter. CI: confidence interval.									

In summary, this is the first study to look at the possible involvement of AKT isoforms in prognosis and diagnosis of lung cancer. The current study also explored the association between mRNA levels of *AKT* isoforms and OS of lung cancer patients with respect to their smoking history, staging of cancer, and sex. The inhibition of the nicotine-activated AKT isoforms pathway may aid in the development of innovative therapeutic techniques for the prevention and treatment of metastatic tumors from smoking-caused lung cancer, which may improve the survival outcome of the patients. Despite the increasing amount of evidence on the altered AKT expression in lung cancer, there is a lack of substantial evidence linking the expression of various AKT isoforms to cancer. The interaction of the selected proteins offers an important information about the evolution of NSCLC and the mechanisms behind cancer incidence, development, metastasis, and drug resistance. The mechanisms behind the actions of these genes must also be investigated, which may assist in the delineation of the predicted network of those genes.

Yours Sincerely,Sahil Khurana^1^, Ajay Pal Singh^2^, Ashok Kumar^3^, Rajeev Nema^4,✉^ ^1^All India Institute of Medical Sciences Bhopal, Bhopal, Madhya Pradesh 462021, India;^2^Department of Medicine, All India Institute of Medical Sciences Rishikesh, Rishikesh, Uttarakhand 249201, India;^3^Department of Biochemistry, All India Institute of Medical Sciences Bhopal, Bhopal, Madhya Pradesh 462021, India;^4^Department of Oncology, 3B Blackbio Biotech India Ltd., Bhopal, Madhya Pradesh 462023, India.^✉^Corresponding author: Rajeev Neman, R&D Department of Molecular Diagnostics, 3B Blackbio Biotech India Ltd., 7-C, Industrial Area, Govindpura, Bhopal, Madhya Pradesh 462023, India. Tel/Fax: +91-755-4077847/+91-755-4282659, E-mail: rajeevnema07@gmail.com.

**Table 4:** Correlation between mRNA expression levels of *AKT* isoforms and the overall survival of male and female patients

Isoforms	Male					Female			
	Low (*N*)	High (*N*)	HR (95% CI)	*P*-value		Low (*N*)	High (*N*)	HR (95% CI)	*P*-value
*AKT1*	552	548	1.06 (0.91–1.24)	4.5e−01		358	356	1.26 (1.00–1.59)	5.2e−02
*AKT2*	330	329	1.49 (1.22–1.83)	1.4e−04		187	187	2.08 (1.46–2.95)	2.8e−05
*AKT3*	556	544	1.28 (1.09–1.50)	2.0e−03		358	356	1.34 (1.06–1.69)	1.4e−02
The mRNA levels of *AKT* isoforms were classified into low and high expression groups according to the median value. Hazard ratio (HR) indicates the measure of the magnitude of the difference between the two curves from Kaplan-Meier plotter. CI: confidence interval.									

## References

[b1] 1Clark SB, Alsubait S. Non small cell lung cancer[M]. Treasure Island (FL): StatPearls, 2021.

[2] (2018). The epidemiology of lung cancer. Transl Lung Cancer Res.

[3] (2013). Online survival analysis software to assess the prognostic value of biomarkers using transcriptomic data in non-small-cell lung cancer. PLoS One.

[4] (2020). AKT2 drives cancer progression and is negatively modulated by miR-124 in human lung adenocarcinoma. Respir Res.

